# Revision of the mollisoniid chelicerate(?) *Thelxiope*, with a new species from the middle Cambrian Wheeler Formation of Utah

**DOI:** 10.7717/peerj.8879

**Published:** 2020-04-09

**Authors:** Rudy Lerosey-Aubril, Jacob Skabelund, Javier Ortega-Hernández

**Affiliations:** 1 Department of Organismic and Evolutionary Biology and Museum of Comparative Zoology, Harvard University, Cambridge, MA, USA; 2 Unaffiliated, Wellsville, UT, USA

**Keywords:** Exceptional preservation, Chelicerata, Mollisoniida, Mollisoniidae, Tagmosis, Miaolingian, Tremadocian

## Abstract

The recent re-interpretation of the Lower Palaeozoic euarthropod group *Mollisonia* as belonging to Chelicerata has triggered a renewed interest for the poorly known family Mollisoniidae. In this contribution, we revise the anatomy, taxonomic diversity, and systematics of *Thelxiope*, the sister-taxon of *Mollisonia.* This mollisoniid genus comprises four species, and is characterized by the presence of one cephalic, seven thoracic (one per tergite), and three pygidial long sagittal spines. The type species, *T. palaeothalassia Simonetta & Delle Cave*, is a rare taxon in the Wuliuan Burgess Shale Formation of Canada, which can be recognized by the hypertrophy of a single of its sagittal spines, the posteriomost one. *T. spinosa (Conway Morris & Robison)*–a species originally assigned to a distinct genus ‘*Ecnomocaris*’ herein synonymised with *Thelxiope*–is known from a single specimen found in the Drumian Wheeler Formation of the House Range of Utah. It differs from the type-species in the hypertrophy of both the anteriormost (cephalic) and the posteriormost (third pygidial) sagittal spines. The same Wheeler strata have also yielded a single specimen of a new taxon, *T. holmani* sp. nov., which lacks hypertrophied sagittal spines and features blunt thoracic tergopleural tips. A putative fourth species, referred to *Thelxiope* sp. nov. A, extends the stratigraphical range of *Thelxiope* to the Lower Ordovician (Tremadocian), and its palaeographic range to West Gondwana. Currently under study, this relatively common component of the lower Fezouata Shale fauna is only briefly discussed. Features characterizing the genus *Thelxiope* and its components almost exclusively pertain to the sagittal spines, for the scarcity and inconsistent preservation of the Cambrian materials as-yet available preclude a confident assessment of the variability of other morphological features. The pygidium in *Thelxiope* and *Mollisonia* is not composed of four, but three tergites essentially similar to thoracic ones, except for the lack of articulations.

## Introduction

**Figure 1:**
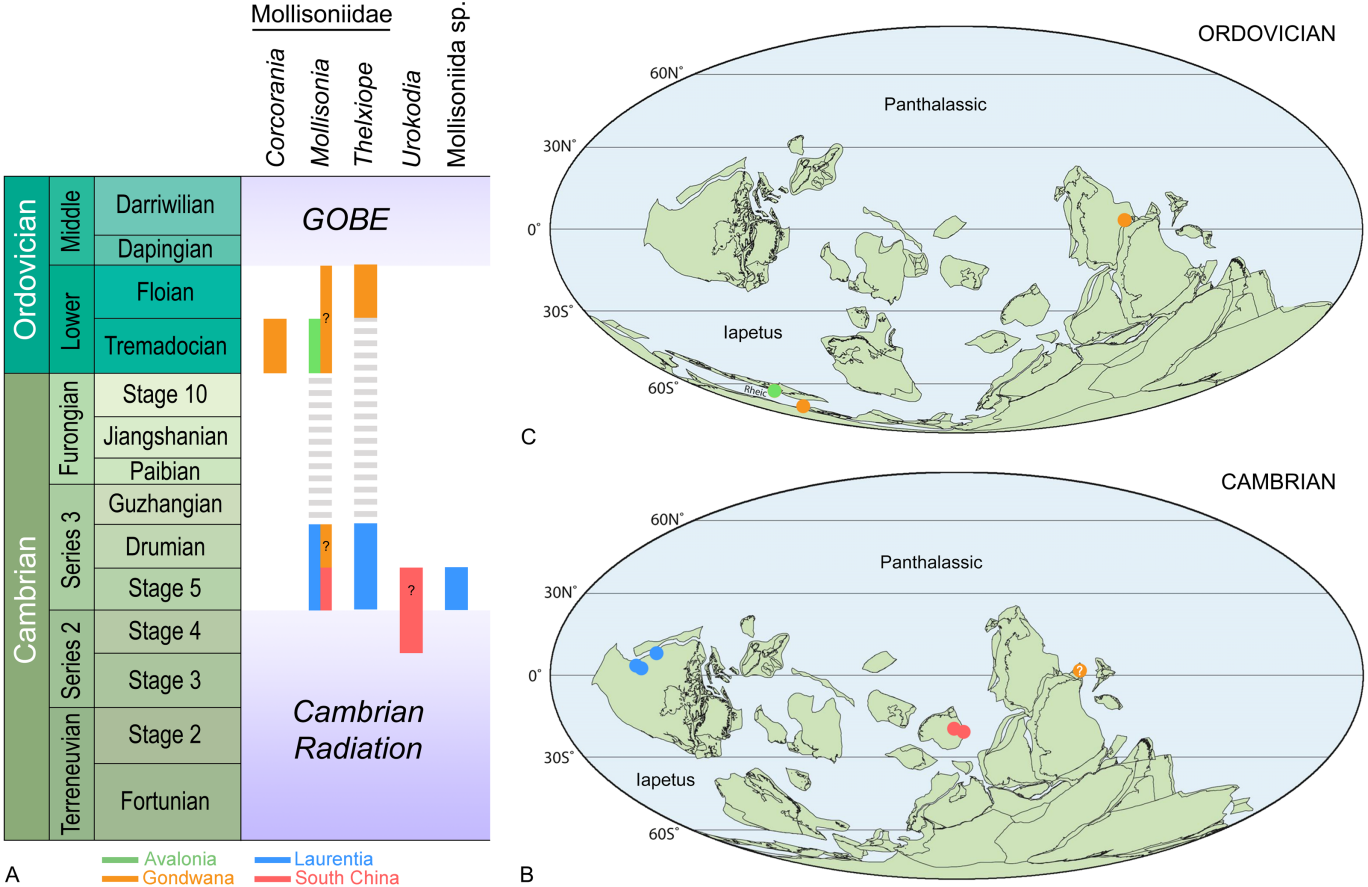
Stratigraphical (A) and palaeogeographical distributions of representatives of the order Mollisoniida during the Cambrian (B) and Ordovician (C). Background maps in (B, C) are from *Torsvik & Cocks (2013)*.

The family Mollisoniidae (*sensu* Lerosey-Aubril et al., in press) is a poorly studied group of Lower Palaeozoic non-biomineralizing euarthropods, with a subparallel-sided body composed of similarly sized cephalic and pygidial shields and a seven-segmented articulated thorax. Although moderately diverse, the family extends from the Miaolingian (Wuliuan) to the Lower Ordovician (Tremadocian) and occurs on four palaeocontinents (Avalonia, East and West Gondwana, Laurentia, and South China —e.g., Walcott, 1912; Jell, 1980; Zhang et al., 2002; Van Roy et al., 2010; Botting et al., 2015). No representatives have been described from older Konservat-Lagerstätten yet, but *Urokodia,* a taxon from the Cambrian Stage 3 Chengjiang biota (Hou, Chen & Lu, 1989; Zhang, Han & Shu, 2002; Hou et al., 2017; see also Zhao et al., 2011, p. 245), might represent a related form with more thoracic segments, hence its recent assignment to the order Mollisoniida (*sensu* Lerosey-Aubril et al., in press) (Figs. 1A–1C). Mollisoniids (i.e., Mollisoniidae, throughout the text) are best known from the Miaolingian strata of Laurentia, with occurrences in the Wuliuan Burgess Shale of Canada (various localities; Walcott, 1912; Walcott, 1931; Simonetta, 1964; Simonetta & Delle Cave, 1975; Caron et al., 2010; Caron et al., 2014; Aria & Caron, 2019), the Wuliuan Spence Shale of northern Utah (Briggs et al., 2008), and the Drumian Wheeler Shale of the House Range and Drum Mountains of western Utah (Robison, 1991; Briggs et al., 2008; Lerosey-Aubril et al., in press). These Laurentian occurrences mostly concern *Mollisonia*, the type genus of the family, in which chelicerae-looking appendages were recently described (Aria & Caron, 2019). If corroborated by further findings, mollisoniids would represent the oldest representatives of the group Chelicerata and as such, would be key to our understanding of the origin of this subphylum of euarthropods.

In the light of the newly acknowledged scientific significance of this group, a taxonomic revision of its members appears both timely and necessary. Lerosey-Aubril et al.’s (in press) formal characterizations of an order Mollisoniida and a family Mollisoniidae provide a systematic framework for that. According to these authors, the family includes four genera: *Corcorania* Jell, 1980, *Ecnomocaris* Conway Morris & Robison, 1988, *Mollisonia* Walcott, 1912 (= *Houghtonites Raymond, 1931*), and *Thelxiope* Simonetta & Delle Cave, 1975. The diagnosis and composition of *Mollisonia* were revised by *Aria & Caron (2019)*, although as acknowledged by these authors a proper taxonomic treatment would require more thorough investigation (see also Lerosey-Aubril et al., in press). The morphology of the Lower Ordovician *Corcorania* is distinct from that of most mollisoniids, at least in the largest adults, which could justify its assignment to a distinct subfamily, rather than a family as proposed by Jell, 1980 (see Lerosey-Aubril et al., in press). Although described 40 years ago, it is reasonably well-known and presently the least in need of taxonomic revision. By contrast, the monospecific *Thelxiope* has never been the subject of a thorough taxonomic study. Our understanding of this taxon is restricted to a short description of its type species (under a different name; see below) provided by Simonetta (1964), and the three specimens he figured, which were later re-illustrated in Simonetta & Delle Cave (1975). In this contribution, we describe a new species of *Thelxiope* from the Drumian Wheeler Formation of Utah, and benefit from this occasion to revise this enigmatic genus, which we regard as a senior synonym of ‘*Ecnomocaris*’. This revision reveals that despite its limited fossil record, this spinose mollisoniid is at least as speciose as *Mollisonia* and exhibits closely comparable geographical and stratigraphical ranges.

## Geological Setting

The palaeogeographical distribution of *Thelxiope* in the Cambrian is restricted to the northern margin of Laurentia (now the western part of North American continent; Fig. 1B), then close to the equator (Torsvik & Cocks, 2013; Torsvik & Cocks, 2017). Representatives of the genus have been recovered from two well-studied Miaolingian Konservat-Lagerstätten: the Burgess Shale in British Columbia (Caron & Rudkin, 2009) and the Wheeler Formation of the House Range in Utah (Robison, Babcock & Gunther, 2015). At that time, the palaeocontinent was rimmed by an expansive carbonate platform, the so-called ‘Great American Carbonate Bank’ (Derby et al., 2012), which separated shallow-water proximal shelf environments from deep-water distal shelf and slope settings. Clastic sediments were deposited on both sides of the carbonate bank, allowing retrospectively the recognition of a series of three lithofacies belts encircling cratonic Laurentia: the inner detrital, middle carbonate, and outer detrital belts (Aitken, 1997).

The Burgess Shale Formation of southeast British Columbia (or ‘thick’ Stephen Formation; Aitken, 1997; Gaines, 2011) was deposited on the seaward margin of the Great American Carbonate Bank, which regionally had formed a prominent relief known as the Cathedral Escarpment (Conway Morris, 1986; Fletcher & Collins, 1998; Gaines, 2011). This position at the foot of a submarine cliff is key to explain the quality of preservation of the exceptionally-preserved biota and its remarkable diversity (Conway Morris, 1986; Gaines, 2014). Indeed, it permitted a rapid and short transport of both the living organisms/fresh organic remains (e.g., recently moulted exoskeletons) and the muddy sediment they lived on, from the normally-oxygenated environments on top of the Cathedral Escarpment down to the deeper-water oxygen-depleted settings of the basin (Gaines, 2014). Thus, the depositional environment of the Burgess Shale fundamentally facilitated both the supply in organic remains and their preservation via a rapid burial under anoxic conditions. The fossil assemblage found in the ‘Phyllopod Bed’ of the Walcott Quarry, including the Burgess mollisoniids discussed in the present contribution, belongs to the *Pagetia bootes* Subzone of the *Bathyuriscus*-*Elrathina* trilobite Zone (Fletcher & Collins, 1998), which corresponds to the *Ptychagnostus praecurrens* agnostoid Zone of North America (Robison & Babcock, 2011). Its taxonomic diversity and ecological structure have been the subjects of thorough investigations (e.g., Conway Morris, 1986; Caron & Jackson, 2008).

The Wheeler strata were most likely deposited closer to the shore compared to the Burgess Shale, although similarly representing shale-dominated deep-water deposits typical of the outer detrital belt. Indeed, these deposits represent the early stage of infilling of a fault-controlled trough that formed a prominent re-entrant within the offshore margin of the carbonate platform during most of the Miaolingian Epoch (Hintze & Robison, 1975; Rees, 1986). Known as the House Range Embayment, this basin is reconstructed as asymmetrical (i.e., a half-graben; Rees, 1986), transitioning to the carbonate bank by a gently sloping ramp to the East (now North), but abruptly separated from it to the West and probably South (now South and East, respectively; Foster & Gaines, 2016). The upper part of the Wheeler Formation has yielded subcontemporaneous, but largely distinct, exceptionally-preserved biotas in the House Range and Drum Mountains of western Utah (Robison, Babcock & Gunther, 2015; Lerosey-Aubril & Skabelund, 2018; Lerosey-Aubril et al., in press), both of early Drumian age according to associated agnostoids (*Ptychagnostus atavus* Zone; Robison & Babcock, 2011). *Thelxiope* exemplifies well this dissimilarity between the two Wheeler faunas, as it is rare but represented by two species in the House Range, and as-yet unreported the Drum Mountains.

## Material and Methods

The material studied consists of the three type specimens of *Thelxiope palaeothalassia* (Simonetta & Delle Cave, 1975) from the Burgess Shale Formation (Walcott Quarry) of British Columbia, and the holotypes of *Thelxiope spinosa* (Conway Morris & Robison, 1988) (formerly ‘*Ecnomocaris’ spinosa;* see below) and *Thelxiope holmani* sp. nov., both from the Wheeler Formation in the House Range of Utah. Details on the geographical and stratigraphical origins of these fossils are provided below, along with a discussion on their preservation and how it may affect the observation of some morphological features. The picture of an additional specimen of *T. palaeothalassia* available on the Royal Ontario Museum’s website dedicated to the Burgess Shale (https://burgess-shale.rom.on.ca/) was also considered when writing the redescription of this taxon. Additionally, high-resolution pictures of the three specimens of ‘*Mollisonia rara*’ (junior synonym of *Mollisonia symmetrica Walcott, 1912*), initially figured by Walcott (1912) and Walcott (1931) and for a time regarded as conspecific with the type specimens of *T. palaeothalassia* (concept of *Parahabelia rara* (Simonetta, 1964); see below), were studied for comparison. All three specimens are from the Burgess Shale and were collected at the Walcott Quarry. Lastly, pictures available on the website of the Yale Peabody Museum and illustrating *Thelxiope* specimens from the Fezouata Shale (Lower Ordovician, Morocco) deposited in its collections were also studied. These specimens likely represent a new species, which is only briefly discussed hereafter as the formal description of this taxon is in progress (P Van Roy, pers. com., 2019).

The specimens were photographed dry or immersed in water, under polarized or cross-polarized illumination, using a Nikon D5500 DSLR fitted with a Nikon 40 mm DX Micro-Nikkor lens or an Olympus DSX110 digital microscope. In the cases of MCZ197957 and USNM424114, images were taken with manual focusing through the focal plane and then stacked using Photoshop CC. Some pictures were mirrored, so that all specimens (or parts of a specimen) are orientated the cephalic shield facing to the left–this facilitates direct comparison between figures. Lastly, Photoshop CC and Inkscape were used to make interpretative drawings out of some of these images and reconstructions of the taxa, and to produce all the figures. Abbreviations used: exs., exsagittally (or parasagittally); sag., mid-sagittally; tr., transverse.

The electronic version of this article in Portable Document Format (PDF) will represent a published work according to the International Commission on Zoological Nomenclature (ICZN), and hence the new names contained in the electronic version are effectively published under that Code from the electronic edition alone. This published work and the nomenclatural acts it contains have been registered in ZooBank, the online registration system for the ICZN. The ZooBank LSIDs (Life Science Identifiers) can be resolved and the associated information viewed through any standard web browser by appending the LSID to the prefix http://zoobank.org/. The LSID for this publication is: urn:lsid:zoobank.org:pub:304BC80B-F898-4842-ACBC-BE3F692CDAE4. The online version of this work is archived and available from the following digital repositories: PeerJ, PubMed Central and CLOCKSS.

## Results

### Systematic Palaeontology

**Table:** 

Phylum EUARTHROPODA Lankester, 1904 (see Ortega-Hernández, 2016)
Subphylum CHELICERATA? Heymons, 1901
Order MOLLISONIIDA Lerosey-Aubril et al., in press
Family MOLLISONIIDAE Lerosey-Aubril et al., in press
Genus *Thelxiope*Simonetta & Delle Cave, 1975

*Emended diagnosis.* Mollisoniids with well-developed sagittal spines on the cephalic shield (one), the thorax (one per tergite), and the pygidium (three), the length of each thoracic one being at least 1.5 the length (sag.) of its corresponding tergite.

*Species included.* The type species *Thelxiope palaeothalassia* Simonetta & Delle Cave, 1975 *T. holmani* sp. nov. (this study); *T. spinosa* (Conway Morris & Robison, 1988); *Thelxiope* sp. nov. A (Van Roy et al., 2010; this study).

*Occurrences.* The Wuliuan Burgess Shale Formation near Mount Field (Walcott Quarry) in British Columbia, Canada; the Drumian Wheeler Formation in the Wheeler Amphitheatre (‘New Dig’ Quarry, ‘U-Dig’ Quarry) of the House Range, Utah, USA; Tremadocian strata of the Fezouata Shale in the Ternata Plain, south-eastern Morocco.

*Remarks*: *Thelxiope* has a complicated taxonomic history. As explained in greater detail below, its name was originally proposed by *Simonetta & Delle Cave (1975)* to replace *Parahabelia* (Simonetta, 1964), a genus distinguished from *Mollisonia* by the presence of ‘dorsal spines’ (‘sagittal spines’ herein). However, neither *Simonetta (1964)* nor *Simonetta & Delle Cave (1975)* explicitly characterized this concept of ‘*Parahabelia*’/*Thelxiope* or proposed a diagnosis for it. To date, the definition of the genus has been regarded as equivalent to that of its type species, *T. palaeothalassia Simonetta & Delle Cave (1975)*, although nowhere acknowledged as so in *Simonetta & Delle Cave (1975)*. The ‘definition’ of the type species solely consists of its description—no list of characters distinguishing it from related forms (i.e., a diagnosis) or comparisons of any kind were included.

**Figure 2:**
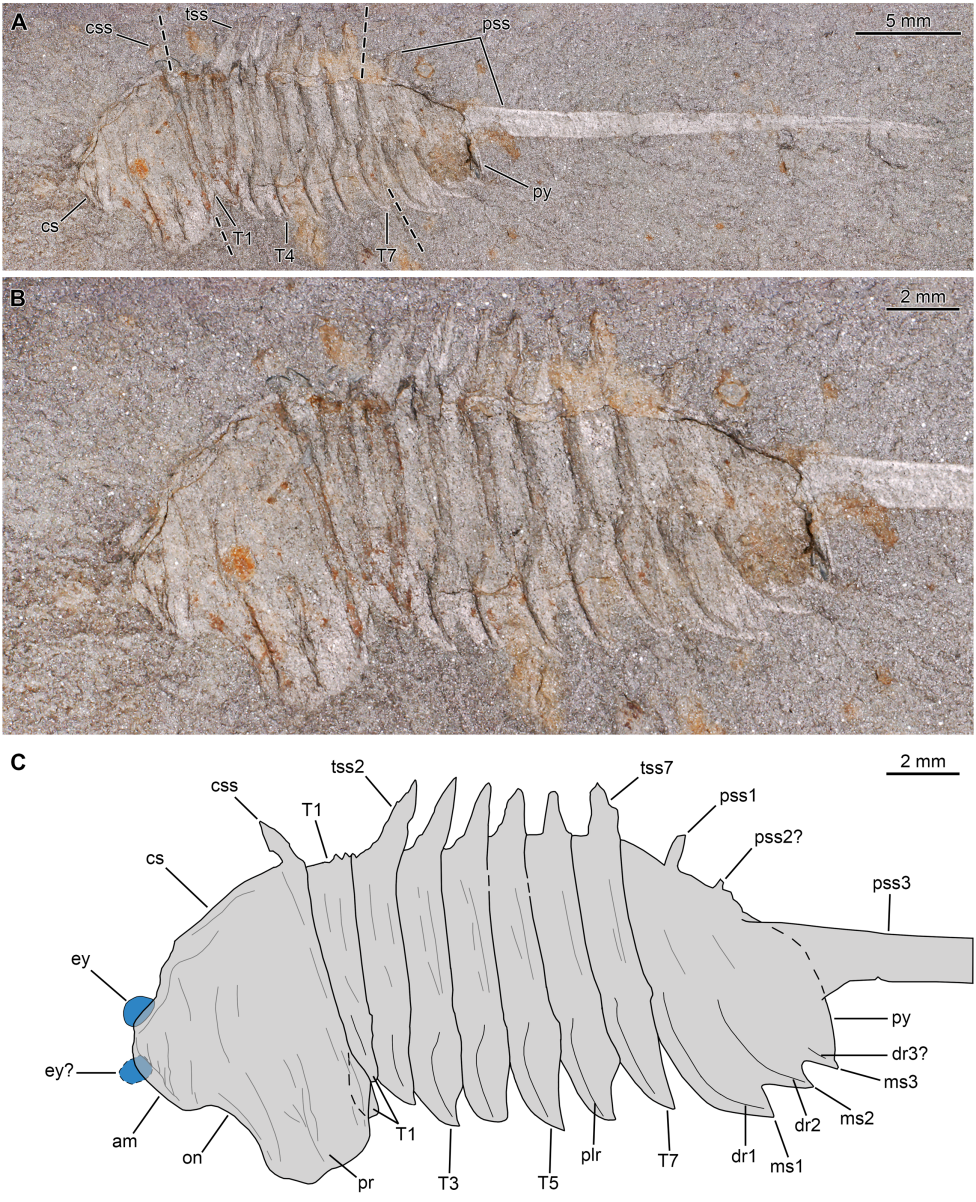
*Thelxiope palaeothalassia*Simonetta & Delle Cave, 1975 from the Cambrian (Wuliuan) Burgess Shale Formation, British Columbia, Canada. (A, B) Holotype specimen USNM144914 (immersed in dilute ethanol, cross polarized light), general (A) and detailed (B) views. (C) Interpretative drawing (credit: Rudy Lerosey-Aubril). Abbreviations: *am*, anterior margin of cephalic shield; *cs*, cephalic shield; *css*, cephalic sagittal spine; *dr*, dorsal ridge; *ey*, eye; *ms*, marginal spine; *on*, optical notch; *plr*, tergopleural ridge; *pr*, tergopleural region; *pss*, pygidial sagittal spine; *py*, pygidium; *T*, thoracic tergite; *tss*, thoracic sagittal spine.

**Figure 3:**
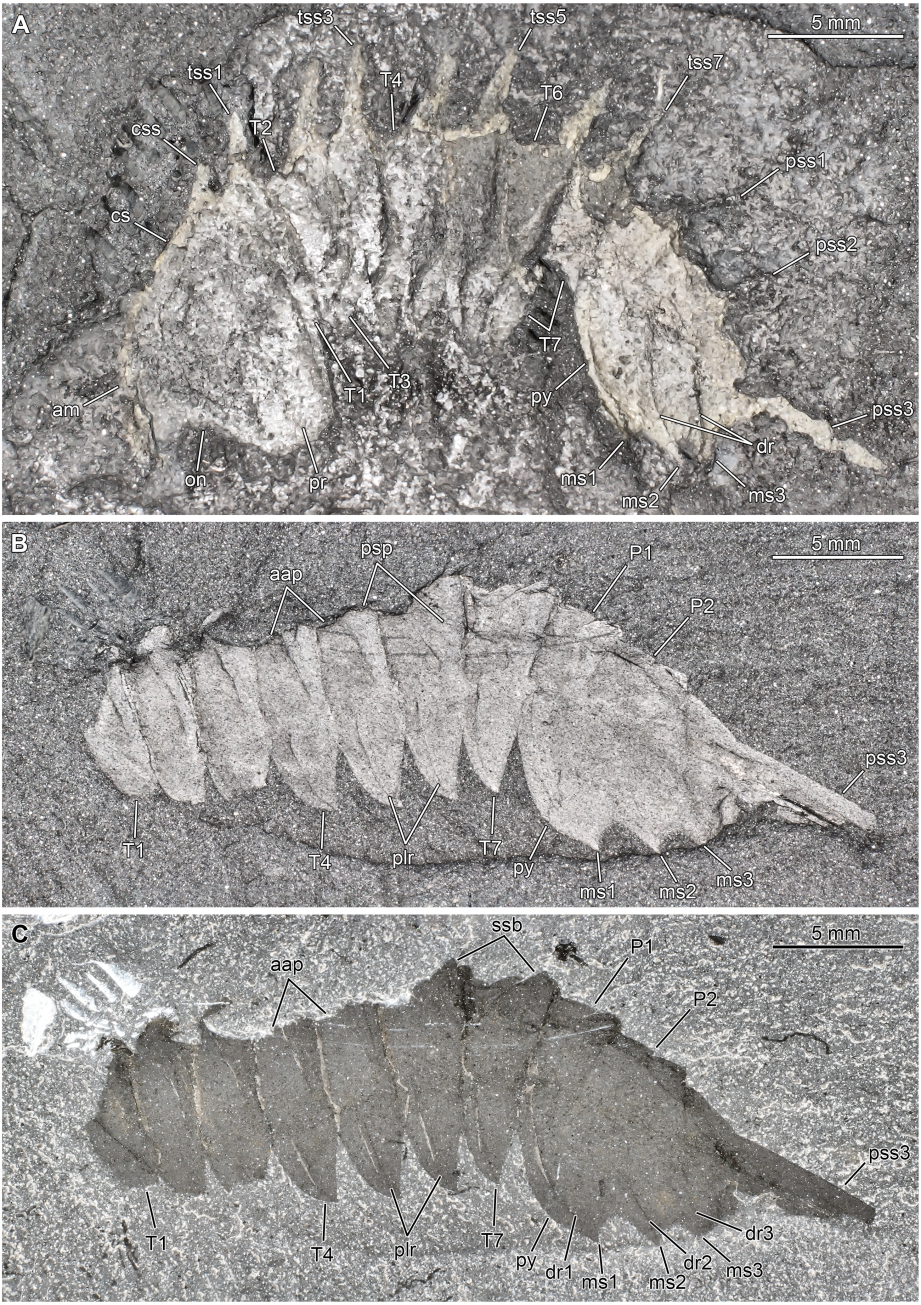
*Thelxiope palaeothalassia*Simonetta & Delle Cave, 1975 from the Cambrian (Wuliuan) Burgess Shale Formation, British Columbia, Canada. (A) Paratype specimen USNM144915 (mirrored; immersed in water, cross polarized light). (B, C) Paratype specimen USNM144916 (mirrored; immersed in water, cross polarized light). Abbreviations: *aap*, anterior articulating part of axial ring; *am*, anterior margin of cephalic shield; *cs*, cephalic shield; *css*, cephalic sagittal spine; *dr*, dorsal ridge; *ms*, marginal spine; *on*, optical notch; *P*, pygidial segment; *plr*, tergopleural ridge; *pr*, tergopleural region; *psp*, posterior swollen part of axial ring; *pss*, pygidial sagittal spine; *py*, pygidium; *T*, thoracic tergite; *tss*, thoracic sagittal spine.

The assignment of two additional species to this genus allows us to propose a simple taxonomic framework for the genus and its representatives. The presence of sagittal spines is a key characteristic of *Thelxiope*, but such spines are also known in *Mollisonia* (e.g., Zhang et al., 2002), hence the considerations of size and distribution in the proposed diagnosis. The sagittal protrusions of *Mollisonia* species are typically small and therefore better regarded as tubercles. This is particularly true in the cephalic region and anterior part of the thorax, where they may even be completely absent (e.g., Lerosey-Aubril et al., in press). By contrast, all *Thelxiope* species display 11 well-developed sagittal spines, including one on the cephalic shield. Within this revised concept of *Thelxiope*, the discrimination between species chiefly relies on the presence and position of one or more hypertrophied sagittal spines, which we characterize as sagittal spines at least twice longer and 50 percent wider (proximally) than the others.

**Table:** 

*Thelxiope palaeothalassia*Simonetta & Delle Cave, 1975
Figs. 2 and 3
1988 *Thelxiope palaeothalassia* Simonetta & Delle Cave; Conway Morris & Robison, pp. 28–30.
In press *Thelxiope palaeothalassia* Simonetta & Delle Cave; Lerosey-Aubril et al.

*Emended diagnosis.* Species of *Thelxiope* characterized by relatively short cephalic, thoracic, and pygidial sagittal spines, except for posteriormost pygidial one that is hypertrophied and exceeds main body length.

*Material, locality, horizon.* The type material consists of three specimens, the holotype (USNM114914; Fig. 2) and two paratypes (USNM114915, 114916; Fig. 3). USNM114914 represents a complete dorsal exoskeleton, USNM114915, an almost complete (most of the hypertrophied pygidial spine is missing), but imperfectly preserved dorsal exoskeleton, and USNM114916, an incomplete dorsal exoskeleton missing the cephalic shield and most of the hypertrophied pygidial spine. We are aware of two additional specimens: one (GSC78462) was mentioned, but not figured by Conway Morris & Robison (1988, p. 29), and the other (GSC74990)—an almost complete dorsal exoskeleton—is illustrated on the Royal Ontario Museum website dedicated to the Burgess Shale. To our knowledge, all these specimens were collected in the Wuliuan strata (*Ptychagnostus praecurrens* Biozone) of the Burgess Shale Formation in the Walcott Quarry (GPS: 51.438486, -116.472228) near Mount Field, British Columbia, Canada. The type specimens are deposited in the collections of the Paleobiology Department of the Smithsonian National Museum of Natural History (prefix USNM), and the additional material in the collections of Geological Survey of Canada (prefix GSC).

*Description*. Cephalic shield representing more than 25 percent of main body length (i.e., spines excluded; sag.), with wide (tr.) and rounded tergopleural regions (Figs. 2,  3A). Anterior margin noticeably convex and flanked laterally by broad ocular notches. Posterior margin straight (Figs. 2B, 2C) to moderately convex (Fig. 3A), extending dorsally into apparently narrow-based, short sagittal spine projecting (mostly) dorsally.

Thorax representing less than half of main body length (sag.) and composed of seven similar articulated tergites (T1–7). Each thoracic tergite with axial region differentiated into two subequal parts: anterior part flat, more (e.g., T3–T5 in Figs. 2B, 2C; T1–T3 in Fig. 3A) or less (T1–7 in Figs. 3B, 3C) concealed under tergite immediately in front (cephalic shield in the case of T1); posterior part swollen (e.g., Figs. 3B, 3C) and giving rise to rather short sagittal spine that projects mostly dorsally (Figs. 2, 3A). Each tergopleural region with convex anterolateral margin meeting concave posterior one to form acuminate distal termination, and bearing marked tergopleural ridge (mostly expressed as a line due to flattening) running subparallel to anterolateral margin proximally and toward tergopleural tip distally (Figs. 2, 3B, 3C). Tergites roughly equal in length (sag.) all along thorax, but moderately increasing in width (tr.) from T1–3 (Figs. 2, 3B, 3C).

Pygidium similar in length (sag.) and width (tr.) to cephalic shield (isopygous condition), and composed of three non-articulated tergites essentially similar in morphology to those of the thorax (Fig. 2). First and especially second pygidial sagittal spines slenderer and shorter than thoracic ones, and projecting dorso-posteriorly (Figs. 2, 3A). Third pygidial sagittal spine hypertrophied, straight, and projecting posteriorly; it is noticeably longer (sag.) than main body (despite possibly being incomplete distally), and is particularly thick proximally, but gently narrows distally to reach half its proximal diameter at (preserved) distal tip (Fig. 2A). Three pairs of weakly-developed marginal spines, which are separated from one another by concave segments of posterolateral margin (Figs. 2 and 3). Three pairs of evenly curved ridges (mostly expressed as lines) running from vicinity of marginal spines toward axial region (Fig. 2B).

Holotype specimen preserves oval eye partially concealed below cephalic shield, but visible along ocular notch of one side (Fig. 2); possible second eye projecting from anterior cephalic margin (i.e., displaced).

*Remarks.* Simonetta (1964) re-assigned the type material of ‘*Mollisonia rara*’ (Walcott, 1912) and three additional specimens he found in the collections of the Smithsonian National Museum of Natural History to a new genus ‘*Parahabelia*’. This decision was motivated by the observation of one or more sagittal spines in the new specimens, which also prompted a redescription of the type species ‘*Parahabelia rara*’ (Walcott, 1912). However, the two specimens (USNM57662, USNM57663) originally described by Walcott (1912) and therefore composing the type material of ‘*P. rara*’ lacked such spines; the same is true of a third specimen (USNM83951) subsequently assigned to this species by Walcott (1931). This was considered as evidence of sexual dimorphism in this species by *Simonetta (1964)*, but *Simonetta & Delle Cave (1975)* disagreed and reinstituted the concept of ‘*Mollisionia rara*’, only to argue that it likely represents a junior synonym of *Mollisonia symmetrica* Walcott, 1912. The latter opinion has been shared by several authors since (Briggs et al., 2008; Aria & Caron, 2019; Lerosey-Aubril et al., in press). The three spine-bearing specimens (USNM114914–114916) were used to create a new genus and a new species, *Thelxiope palaeothalassia* Simonetta & Delle Cave, 1975, the description of which was said to be the same as the one provided by *Simonetta (1964)* for his ‘*Parahabelia rara*’.

Our redescription of *T. palaeothalassia* is more detailed than Simonetta’s (1964) brief account and differs from it in two meaningful ways first pointed out by *Conway Morris & Robison (1988)*. Firstly, the thorax of this species is composed of seven, rather than six freely-articulated tergites. We suspect that the two segments that Simonetta interpreted as ‘incorporated into the cephalon’ actually represent the anteriormost freely-articulated thoracic tergite that is partially tucked under the cephalic shield—the posterior cephalic margin and wrinkles resulting from the compaction of these two superposed skeletal elements might have been misinterpreted as intersegmental furrows (Figs. 2B, 2C). Thus, the organisation of the body in the type species of *Thelxiope* is fundamentally similar to that of all the representatives of the family Mollisoniidae, as characterized by Lerosey-Aubril et al. (in press). Moreover, Simonetta described the pygidium of ‘*P. rara*’ as bearing three short and one long sagittal spines (three ‘dorsal spines’ and one ‘telsonic spine’), while we believe that it possesses only three of such spines like its congeneric species. The second short sagittal spine projects postero-dorsally, whereas the hypertrophied posteriormost one projects posteriorly (Fig. 3A). This, and the poor (Figs. 2, 3A) or absent (Figs. 3B, 3C) preservation of the smaller pygidial sagittal spines in the available specimens, might have misled Simonetta into hypothesizing the presence of an additional short spine between the second sagittal spine and the hypertrophied ‘telsonic’ one. There is no clear evidence of a non-segmented terminal piece in the pygidium (‘fused telson’ of Simonetta, 1964) of *Thelxiope* at all, and if present it would be confined to a small area extending from the pygidial posterior margin to the third sagittal spine.

*T. palaeothalassia* is easily distinguished from other species of *Thelxiope* by the presence of a single hypertrophied sagittal spine (the posteriormost pygidial one), the moderate length of its other sagittal spines, the differentiation of thoracic axial ‘rings’ into flat anterior and swollen posterior parts, and particularly well-expressed tergopleural lines/ridges on the thoracic tergites and the pygidium. The type species also differs from *T. holmani* sp. nov. by the presence of narrow-based, dorsally-projecting sagittal spines and acute tergopleural terminations in the thorax.

**Table:** 

*Thelxiope holmani* sp. nov.
urn:lsid:zoobank.org:act:097471F6-DB92-4356-BF02-C50EFA363808

*Diagnosis.* Species of *Thelxiope* with broad-based sagittal spines, none hypertrophied, and straight to rounded thoracic tergopleural tips.

*Etymology.* In honour of Clayton Holman, the discoverer of the holotype.

*Material, locality, horizon.* The type material consists of the sole holotype (MCZ197957), a laterally compressed, complete dorsal exoskeleton, which is 52 mm long along its dorsal margin (spines excluded). This specimen was collected in the Drumian strata (*Ptychagnostus atavus* Biozone) of the upper Wheeler Formation at the ‘New Dig Quarry’ (GPS: 39.35883333, -113.27861111) in the House Range, Millard County, Utah. The specimen is housed in the Invertebrate Paleontology collections at the Museum of Comparative Zoology at Harvard University (prefix MCZ).

**Figure 4:**
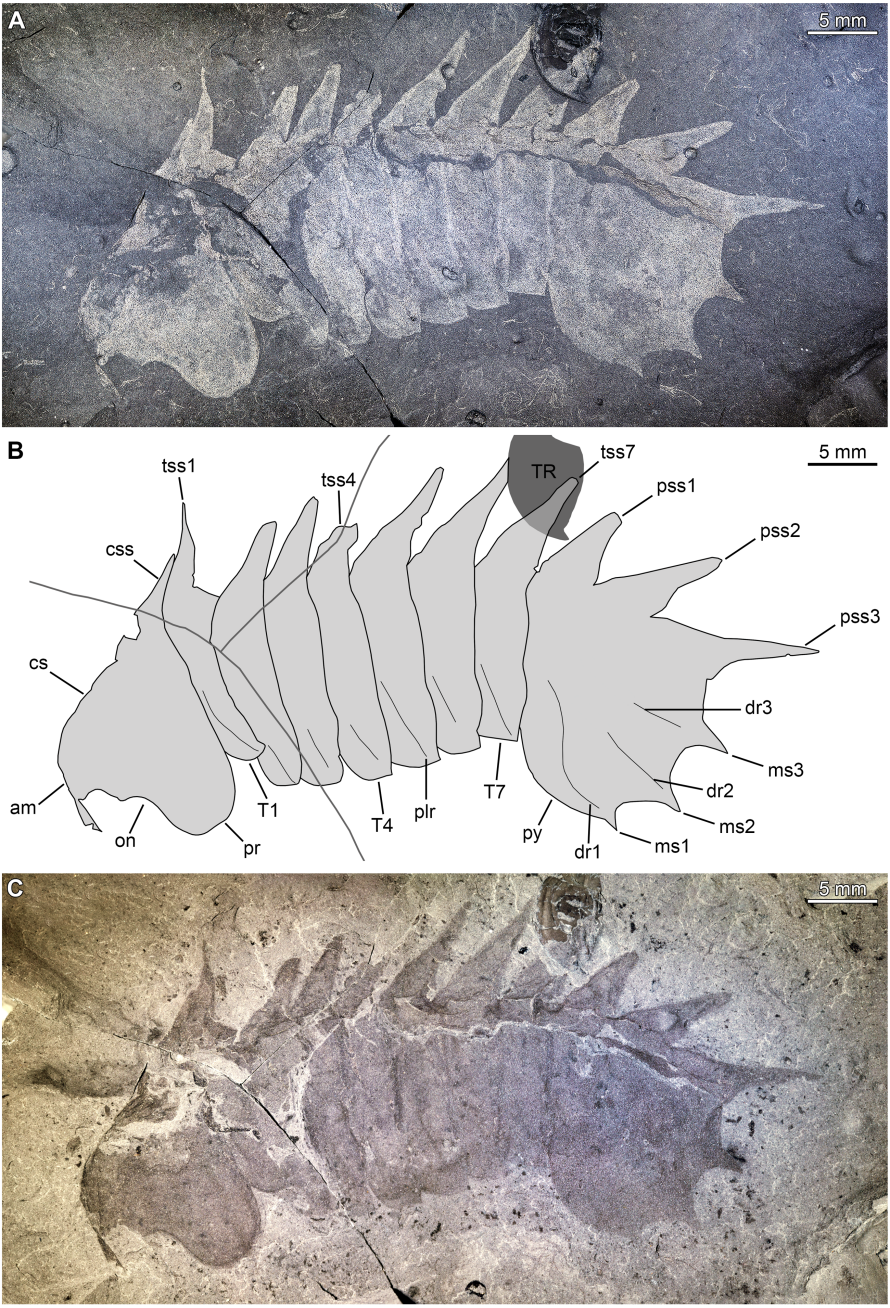
*Thelxiope holmani* sp. nov. from the Cambrian (Drumian) Wheeler Formation in the House Range of Utah, USA. Holotype specimen MCZ197957. (A, C) General views of specimen immersed in dilute ethanol using cross polarized light. (B) Interpretative drawing (credit: Rudy Lerosey-Aubril). Abbreviations: *am*, anterior margin of cephalic shield; *cs*, cephalic shield; *css*, cephalic sagittal spine; *dr*, dorsal ridge; *ms*, marginal spine; *on*, optical notch; *plr*, tergopleural ridge; *pr*, tergopleural region; *pss*, pygidial sagittal spine; *py*, pygidium; *T*, thoracic tergite; *TR*, trilobite; *tss*, thoracic sagittal spine.

*Description*. Cephalic shield less than a fifth of main body length (sag.), with wide (tr.) and rounded tergopleural regions (Figs. 4A–4C). Anterior margin slightly convex and flanked laterally by broad ocular notches. Posterior margin slightly concave, extending dorsally into broad-based sagittal spine that rapidly thins distally and projects posterodorsally. Thorax composed of seven articulated tergites and representing about half of main body length (sag.). T1 notably narrower (tr.) than cephalic shield and T2, due to shorter (tr.) tergopleural extent (Figs. 4A–4C). Tergopleurae with straight lateral margins subparallel to sagittal axis, which form rounded angles with anterior margins and acute ones with posterior margins. Tergopleural ridge (expressed as line in flattened specimen) posteriorly curved distally and reaching lateral margin close to meeting point with posterior margin. Whole exposed part of tergite axis extends dorsally into broad-based sagittal spine, the posterior margin of which abruptly changes from dorsal to anterodorsal orientation distally, suggesting possible post-mortem deformation. T2–T7 are essentially similar in length (sag.), but their width (tr.) slightly increases from T2 to T3, then progressively and moderately decreases from T3 to T7. Their tergopleurae are wider (tr.) than that of T1 but similar in outline, and each bears tergopleural ridge (expressed as line due to flattening) running towards postero-lateral angle. Sagittal spines increasingly robust and posterodorsally directed from T2 to T7; each arises from the whole exposed part of tergite axis and is therefore broad-based, although abruptly thinning distally. Pygidium as wide (tr.) as cephalic shield, but longer than it (moderate macropygous condition), representing almost a third of main body length (sag.) (Figs. 4A–4C). Three pairs of well-defined marginal spines separated by concave segments of posterolateral margin, and continued dorsally by thin sigmoidal ridges (represented by faint lines in flattened holotype) that run antero-medially. Three broad-based sagittal spines projecting dorsally and increasingly posteriorly, with posteriormost one being somewhat slenderer than others distally and seemingly merging with posterior pygidial margin posteriorly.

*Remarks.* MCZ197957 is sufficiently well-preserved and complete to rule out its assignment to *Thelxiope palaeothalassia* or *T. spinosa*, thus warranting the creation of a new species. *T. holmani* sp. nov. differs from congeneric species in the lack of cephalic or pygidial hypertrophied sagittal spine, and the broader insertions of its thoracic sagittal spines (full tergite length, rather than posterior halves only) and their more posterior orientation. All exhibit a similar morphology, with a broad base, and an abrupt narrowing followed by a progressive tapering distally, which rules out the interpretation of one (or more) of them being an incompletely preserved hypertrophied spine. *T. holmani* sp. nov. is further differentiated from the type species by the straight to slightly rounded, rather than acute tips of its thoracic tergopleurae, a character unclear in *T. spinosa*.

**Table:** 

*Thelxiope spinosa* (Conway Morris & Robison, 1988)
Figs. 5 and 6
1988 *Ecnomocaris spinosa Conway Morris & Robison*, pp. 27–30: fig. 19, 20.
1991 *Ecnomocaris spinosa Conway Morris & Robison*, p. 86: fig. 7.
2008 *Ecnomocaris spinosa*; Hendricks, Lieberman & Stigall: tab. 1.
2015 *Ecnomocaris spinosa Conway Morris & Robison*; Robison, Babcock & Gunther, p. 94.
2016 *Ecnomocaris spinosa Conway Morris & Robison*; Foster & Gaines, pp. 299–300.
In press *Ecnomocaris spinosa Conway Morris & Robison*; Lerosey-Aubril et al.

*Diagnosis*. Species of *Thelxiope* characterized by the hypertrophy of the cephalic sagittal spine, and to a lesser extent, its posteriormost pygidial sagittal spine.

**Figure 5:**
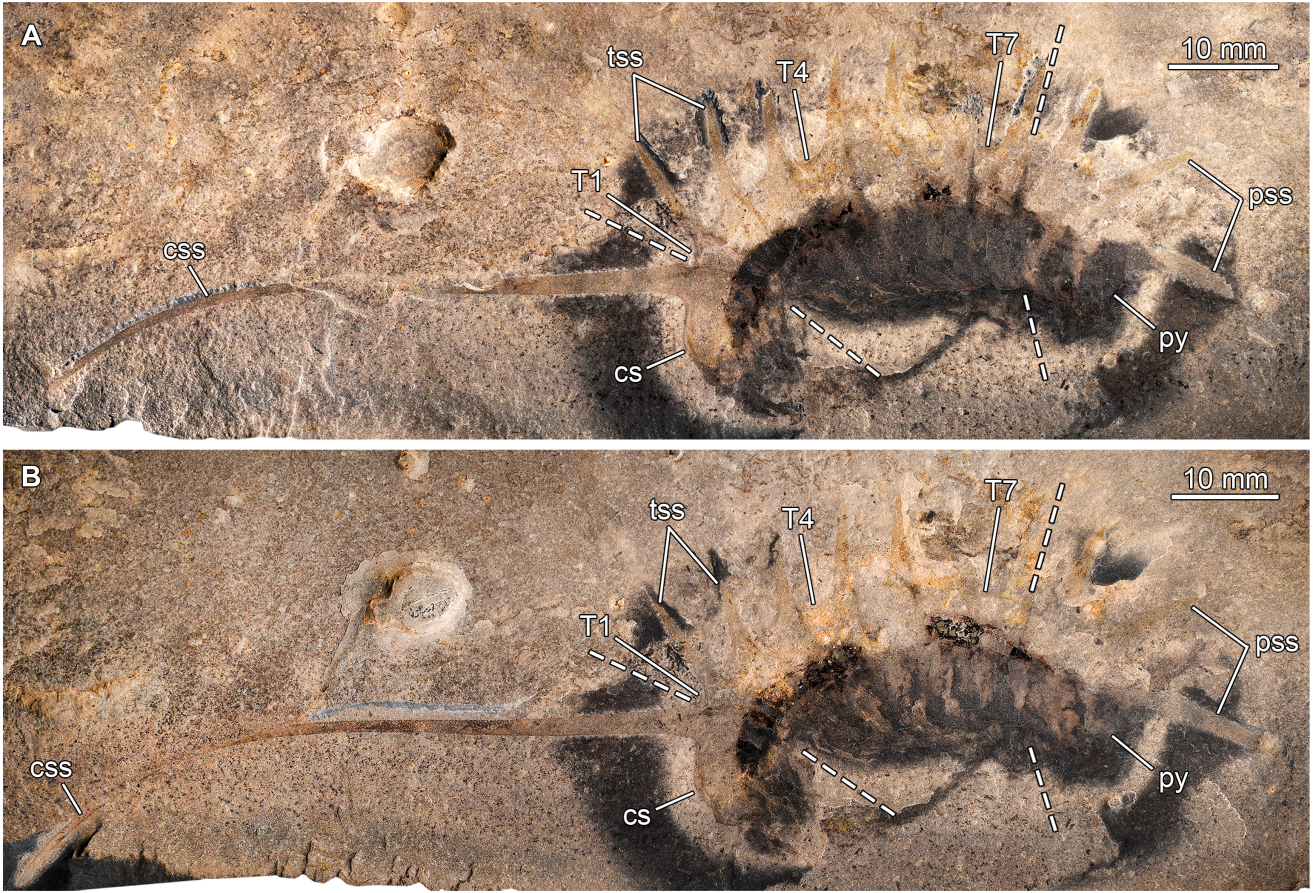
*Thelxiope spinosa* (Conway Morris & Robison, 1988) from the Cambrian (Drumian) Wheeler Formation in the House Range of Utah, USA. (A, B) General views of holotype specimen USNM424114 (immersed in dilute ethanol; cross polarized light; photo credit: Sarah Losso). (A) Part. (B) Counterpart (mirrored). Abbreviations: *cs*, cephalic shield; *css*, cephalic sagittal spine; *pss*, pygidial sagittal spines; *py*, pygidium; *T*, thoracic tergite; *tss*, thoracic sagittal spines.

**Figure 6:**
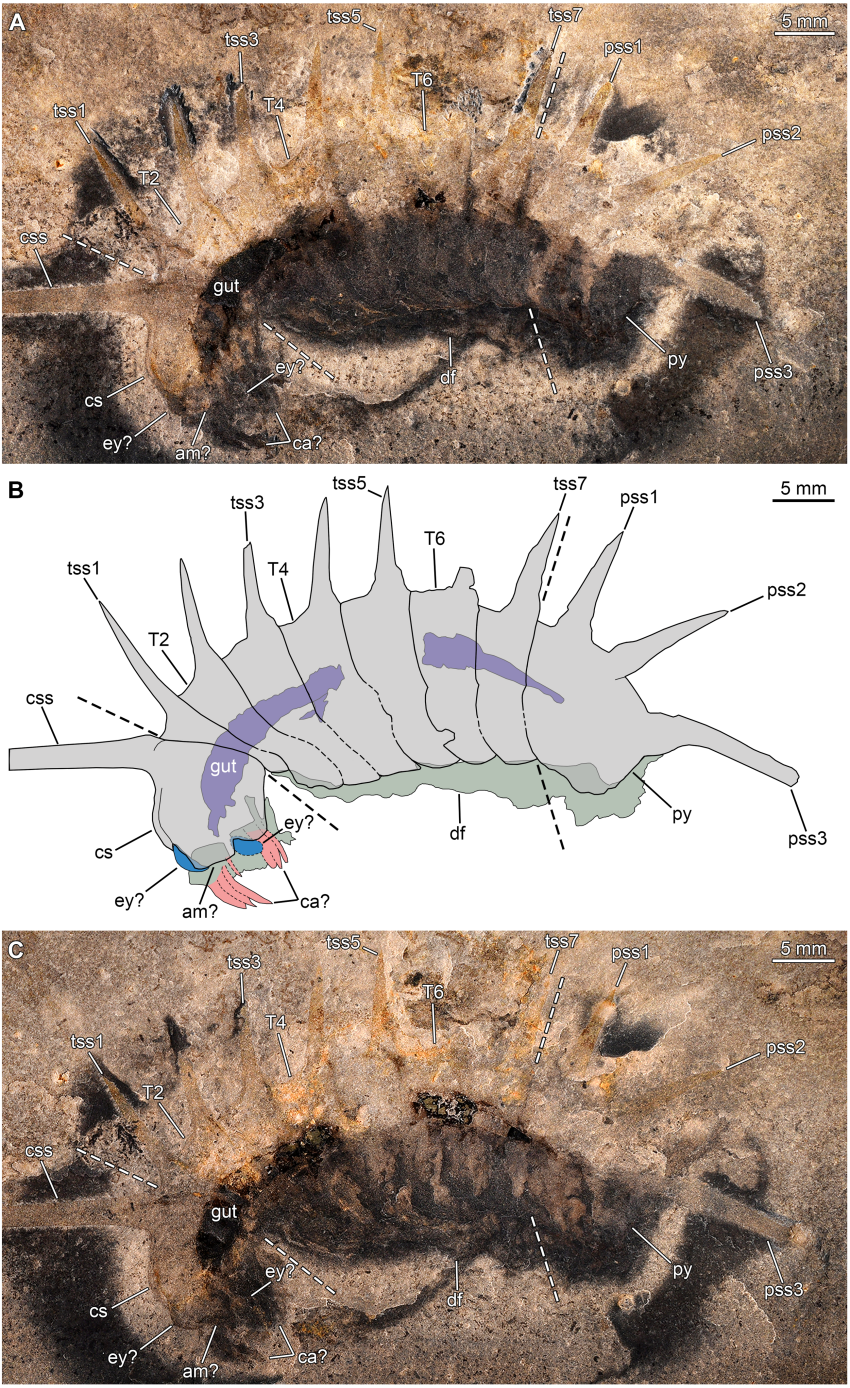
*Thelxiope spinosa* (Conway Morris & Robison, 1988) from the Cambrian (Drumian) Wheeler Formation in the House Range of Utah, USA. (A, C) Details views of holotype specimen USNM424114 (immersed in dilute ethanol, cross polarized light; photo credit: Sarah Losso). (A) Part. (C) Counterpart (mirrored). (B) Interpretative drawing combining details of both parts (credit: Rudy Lerosey-Aubril). Abbreviations: *am*, anterior margin of cephalic shield; *ca*, cephalic appendages; *cs*, cephalic shield; *css*, cephalic sagittal spine; *df*, decay fluid; *ey*, eye; *pss*, pygidial sagittal spine; *py*, pygidium; *T*, thoracic tergite; *tss*, thoracic sagittal spine.

*Material, locality, horizon.* The type material solely consists of the holotype, the part and counterpart of a complete dorsal exoskeleton with possible poorly-preserved appendages and eyes (Figs. 5 and 6). The specimen was collected in the Drumian strata (*Ptychagnostus atavus* Biozone) of the upper Wheeler Formation at the type locality (now ‘U-Dig quarry’; GPS: 39.354429, -113.278776) in the House Range, Millard County, Utah. It is housed in the collections of the Department of Paleobiology of the Smithsonian National Museum of Natural History (USNM424114).

*Redescription of the holotype*. USNM424114 is interpreted as having been flattened in an oblique orientation anteriorly (cephalic shield and anterior thoracic segments) and in a lateral orientation posteriorly. Cephalic shield representing about a sixth of main body length, and tilted ventrally (Figs. 5A, 5B, 6A–6C). Anterior margin hardly discernible, but apparently flanked by pair of moderately-incised ocular notches (Figs. 6A–6C). Cephalic sagittal spine extremely long, slightly exceeding main body length (sag.), and projecting dorsally and to a lesser extent anteriorly (Figs. 5A, 5B). It is broad and straight proximally, but progressively becomes thinner and increasingly bent forwards distally. Thorax represent c. 60 percent of main body length (sag.; Figs. 6A–6C). T1 conspicuously reduced in both width (tr.) and length (sag.). Its sagittal spine is long (c. three times the sagittal length of T1), slender, and pointy. It projects dorsally and slightly posteriorly from exposed posterior half (sag.) of tergite. T2 wider (tr.) and a little longer (sag.), but otherwise similar to T1. T3–T7 similar to T2, albeit a little longer (sag.), bearing increasingly stouter and slightly shorter sagittal spines; morphology of their tergopleural tips unknown due to poor preservation. Pygidium slightly larger than cephalic shield (moderate macropygous condition), its length c. a fourth of main body length (sag.). Presence of marginal spines and corresponding dorsal ridges unknown due to poor preservation (Figs. 6A–6C). Second sagittal spine only a little longer and stouter than anteriormost one, and notably slenderer than third one. Preserved part of this posteriormost sagittal spine broad and only moderately narrowing distally (Figs. 5 and 6), suggesting that it originally was much longer than any other trunk sagittal spines (i.e., hypertrophied).

Elongate central structure composed of thick layer of dark dull material, and running under posterior two-thirds of cephalic shield, thoracic tergites (except T5), and anterior third of pygidium, is interpreted as incomplete digestive tract (Figs. 6A–6C). It is large anteriorly (c. a third of cephalic width, tr.)—except for short, much narrower anteriormost part that possibly represents the oesophagus—and progressively tapers posteriorly. Posteriormost portion of gut tract apparently missing. Dorsal exoskeleton ventrally fringed by poorly-delimitated dark stain interpreted as appendages at advanced stage of decay surrounded by microbial film. Two ovoid structures abutting the cephalic shield are regarded as possible eyes, and two sets of elongate darker structures are tentatively interpreted as cephalic appendages (Figs. 6B–6C).

*Remarks.* Our redescription of UMNH.IP.6162 is less conservative, but largely congruent with the original description of *Conway Morris & Robison (1988)*. In addition to the fact that the cephalic sagittal spine appears to us complete in the counterpart, a careful examination revealed that the boundaries between the dorsal sclerites are expressed, at least to some extent, in both part and counterpart, especially dorsally. This allows a more precise reconstruction of the exoskeletal morphology of this taxon, although some features remain insufficiently known, such as the presence of pygidial dorsal ridges and marginal spines, or the shape of thoracic tergopleural tips. We also believe that some structures can be recognized, even if tentatively, in the vicinity of the cephalic shield. An ovoid structure, consistent in size (relative to the cephalic shield) and shape with an eye (Aria & Caron, 2019), is rather well-preserved on both parts. A second eye might be represented by a particularly dark, ovoid area on the other side of the cephalic shield (left side if specimen orientated as in Figs. 5 and 6). Likewise, the dark halo fringing the ventral margin of the cephalic shield extends into two main sets of three darker digitiform areas each, consistent in size and orientation with cephalic appendages projecting from under the head (Aria & Caron, 2019).

This more comprehensive interpretation of UMNH.IP.6162 does not reveal any features that would to us warrant the assignment of this species to a distinct genus ‘*Ecnomocaris*’. As acknowledged by *Conway Morris & Robison (1988)*, most aspects of the morphology of this fossil are similar to *T. palaeothalassia*, and our restudy of the type materials of both taxa confirms this view. The basic organization of the body into a cephalic shield, a seven-segmented thorax, and a large three-segmented pygidium, and the presence of sagittal protrusions point to affinities with the Mollisoniidae (Lerosey-Aubril et al., in press), while the great development of the sagittal protrusions into spines is diagnostic of the genus *Thelxiope* (as redefined herein) within this family. Accordingly, we propose to regard the type species of ‘*Ecnomocaris*’ as a distinct species of *Thelxiope*, *T. spinosa* (Conway Morris & Robison, 1988), and to consider the former genus as a junior synonym of the latter.

*T. spinosa* is easily distinguished from congeneric species by the spectacular development of its cephalic sagittal spine. Its thoracic sagittal spines are also longer than those of the type species. They essentially project dorsally and have narrow bases, unlike those of *T. holmani* sp. nov. The presence of a hypertrophied posteriormost sagittal spine in the pygidium further distinguishes *T. spinosa* from the new Wheeler species.

**Table:** 

*Thelxiope* sp. nov. A

*Material, locality, horizon.* One specimen (YPM-IP226544), a complete dorsal exoskeleton, was illustrated in Van Roy et al. (2010, fig. 1F); this and about 45 others are deposited in the collections of Invertebrate Paleontology of the Yale University Peabody Museum. Most of these specimens were collected from the Tremadocian part (*Araneograptus murrayi* graptolite Zone; Lefebvre et al., 2018) of the Fezouata Shale in a quarry between Ezegzaou and Bou Glf, Ternata Plain, north of Zagora, southeastearn Morocco.

**Figure 7:**
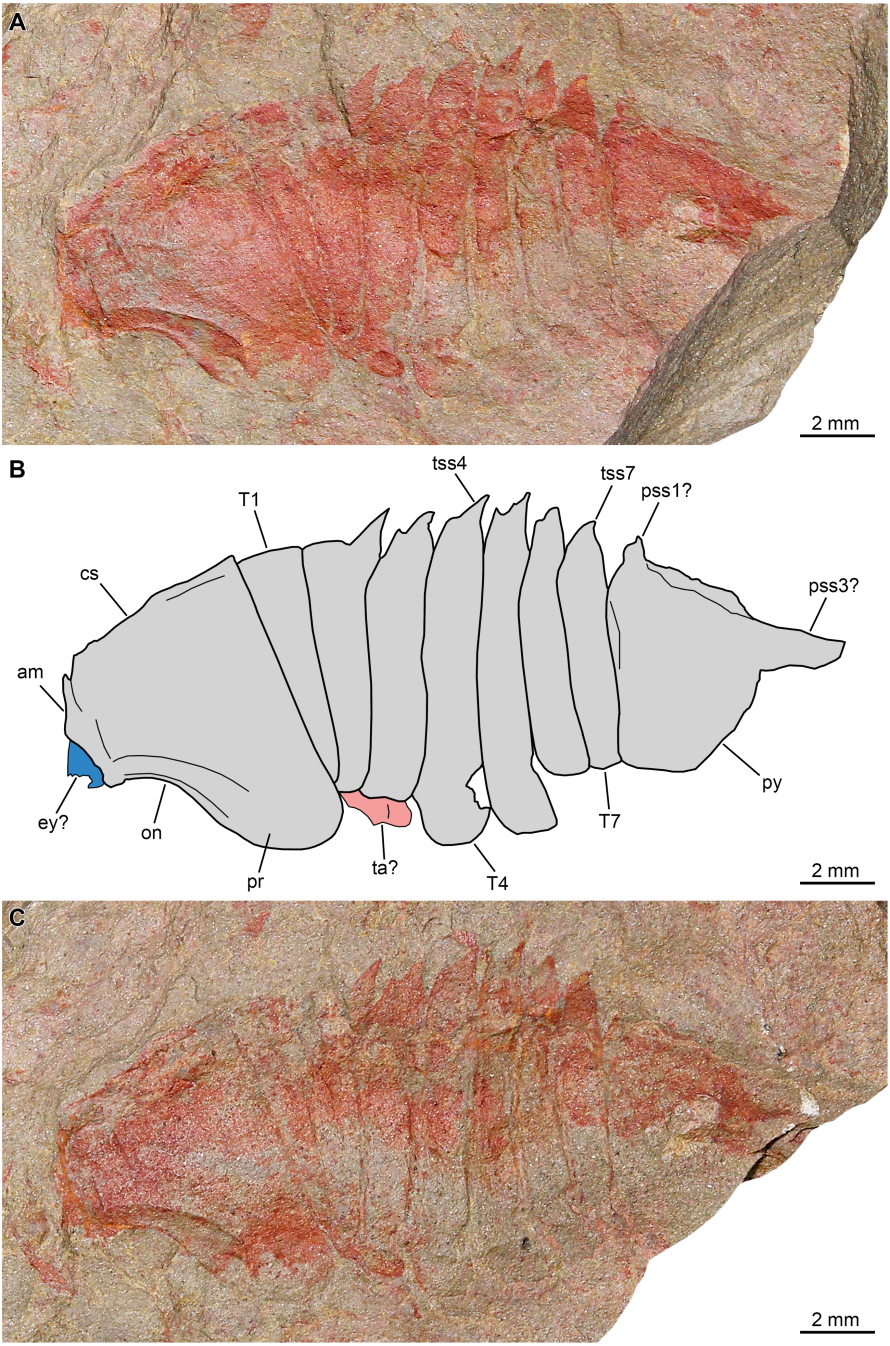
*Thelxiope* sp. nov. A from the Ordovician (Tremadocian) lower Fezouata Shale in the Ternata Plain of southeastern Morocco. YPM-IP226544, almost complete dorsal exoskeleton, preserved flattened (mostly) laterally. (A, C) General views of part (A) and counterpart (B; mirrored) (photo credit: Jessica Utrup). (B) Interpretative drawing (credit: Rudy Lerosey-Aubril). Abbreviations: *am*, anterior margin of cephalic shield; *cs*, cephalic shield; *ey*, eye; *on*, optical notch; *pr*, tergopleural region; *pss*, pygidial sagittal spine; *py*, pygidium; *T*, thoracic tergite; *ta*, trunk appendage; *tss*, thoracic sagittal spine.

*Remarks*. This material is currently under investigation (P Van Roy, pers. com., 2019) and therefore, our brief discussion focuses on the specimen previously published by Van Roy et al. (2010, fig. 1F). YPM-IP226544 (Figs. 7A–7C) shows a series of well-developed, but incomplete sagittal spines on T2–T7, which justifies its assignment to *Thelxiope*. The pygidium is incomplete, but it seems to bear a small sagittal spine close to its anterior margin and a hypertrophied one projecting posteriorly from its posteriormost region. The quality of preservation of the specimen does not allow to ascertain with confidence whether the absence of sagittal spines on the cephalic shield and T1 is original or the result of post-mortem alteration. The thoracic spines are broad-based like those of *T. holmani* (Fig. 3), but they are short relative to the length (sag.) of the corresponding tergites, which might be due to breakage (several show blunt and irregular terminations). Otherwise, they essentially project dorsally, resembling those of *T. palaeothalassia* (Figs. 2, 3A). This Moroccan species also differs from *T. holmani* by the presence of a hypertrophied posteriormost pygidial spine. Both the length (sag., exs.) and width (tr.) of the thoracic tergites noticeably increase from T1–T4 and decrease from T5–T7, T4 and T5 being wider (tr.) than the cephalic shield (Figs. 7A–7C), a feature never observed in any of the three Cambrian species. This unique combination of characters suggest that this Gondwanan fossil represents a distinct species of *Thelxiope*, the only one known outside of the Miaolingian strata of Laurentia (Fig. 1).

## Discussion

### Taxonomic significance of spinosity pattern

**Figure 8:**
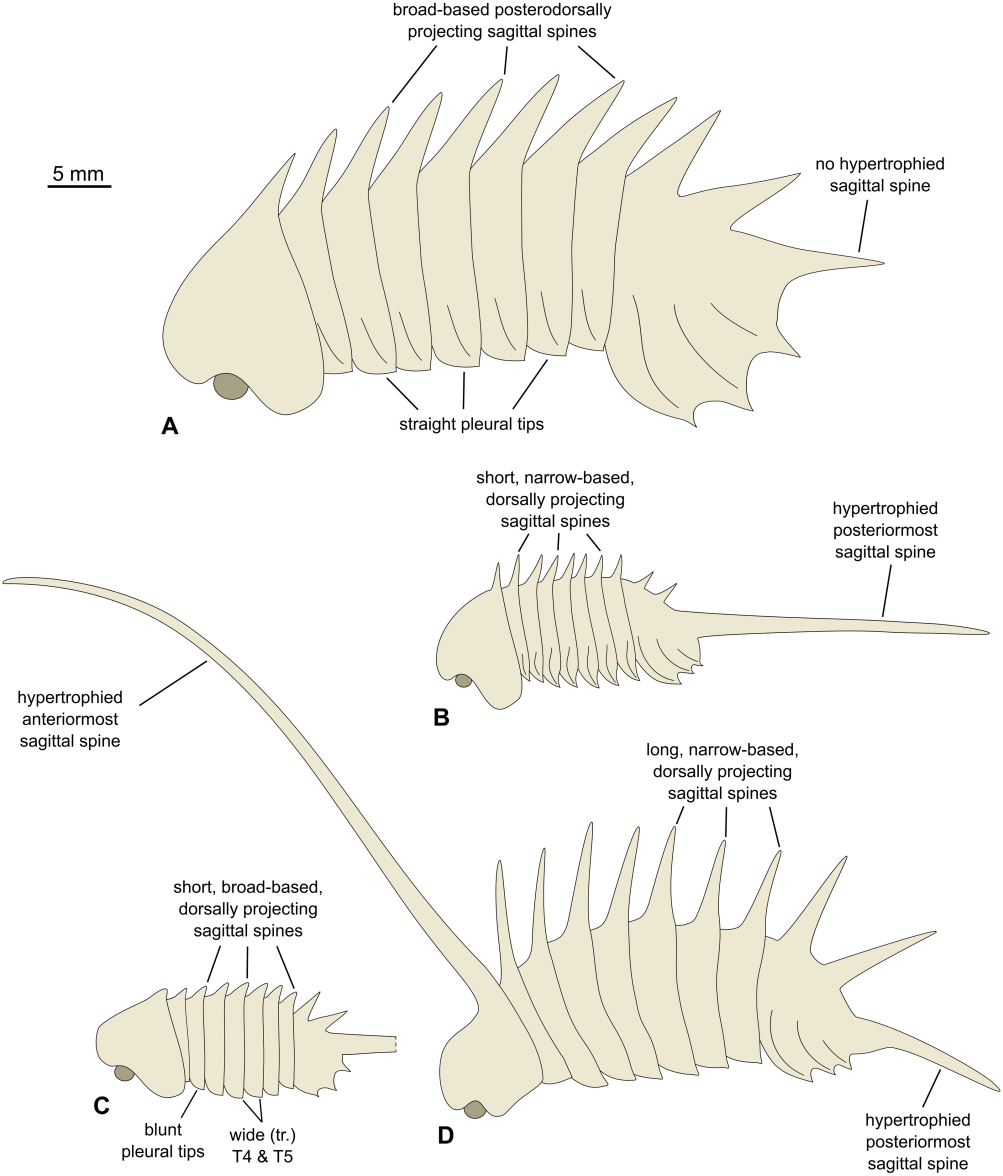
Morphological reconstructions of the four species of *Thelxiope* and their diagnostic features. (A) *Thelxiope holmani* sp. nov., Drumian Wheeler Shale. (B) *Thelxiope palaeothalassia* (type species), Wuliuan Burgess Shale. (C) *Thelxiope* sp. nov. A, Tremadocian, lower Fezouata Shale. (D) *Thelxiope spinosa*, Drumian Wheeler Shale. Note that the morphology of the lateral margins of the dorsal exoskeleton in *T. spinosa*, and the presence of marginal and some sagittal spines on the cephalic shield and pygidium of *Thelxiope* sp. nov. A have been extrapolated from congeneric species. Credit for all drawings: Javier Ortega-Hernández.

Our revised concept of *Thelxiope* emphasizes the presence of 11 well-developed sagittal spines on the dorsal exoskeleton, which are distributed from the posterior part of the cephalic shield to the posterior tip of the pygidium. Whether these spines are broad- or narrow-based, dorsally or dorso-posteriorly directed and more importantly, which of them (if any) are hypertrophied constitute the main features used to differentiate the species assigned to this genus (Figs. 8A–8C). The shape of the distal terminations of the thoracic tergopleurae (acute or straight) is the only feature non-related to sagittal spines used in the diagnosis of one of the species.

The prominence of the characters pertaining to the sagittal spines is justified by the fact that they are more easily and reliably observed than those concerning other aspects of the exoskeletal morphology of *Thelxiope* species. In all of these taxa, but the insufficiently known *Thelxiope* sp. nov. A, the number and segmental distribution of the sagittal spines is the same, which greatly facilitates the identification of a missing one in an incomplete or imperfectly preserved specimen. Likewise, recognizing that a spine is hypertrophied is fairly easy, even when this spine is incompletely exposed or broken. Hypertrophied spines are not only much longer than normal sagittal spines, but also much wider proximally. This is how we can infer that the broken posteriormost sagittal spine of *T. spinosa* likely represents a second hypertrophied spine, or that the absence of a hypertrophied in *T. holmani* is original. The number and position of these hypertrophied spines are regarded as robust diagnostic characters, because it is particularly unlikely that taphonomy could affect their appearance and not that of the regular spines without being noticed. An incompletely preserved or exposed spine, whether hypertrophied or not, typically exhibits a blunt and/or irregular, rather than acute termination. When such incomplete spines are discarded, it appears that regular sagittal spines exhibit limited size and shape variations within a specimen or more importantly within a given species; this allows the use of their characteristics in the diagnoses proposed above. For instance, the lengths of these spines relative to the body in *T. palaeothalassia* are similar in all the specimens exhibiting them, and noticeably much smaller than those of *T. holmani* and *T. spinosa*. Similarly, all the sagittal spines of *T. holmani* are broad-based, whereas all the non-hypertrophied spines of the specimens belonging to other species are widely-spaced proximally. The latter feature cannot be explained by incomplete exposure, especially in *T. spinosa* where the regular sagittal spines are longer than in *T. holmani*. Thus, spine-related characters are preferentially used in our diagnoses for two main reasons: (1) they are repeatedly observed within a given individual and/or the individuals of a given species; (2) impact of incomplete preservation or exposure on them is easily detectable.

Our understanding of the morphological variability of these taxa is otherwise severely impeded by the scarcity and inconsistent preservation of the available material. For instance, *T. palaeothalassia* is known from five specimens only, making it one of the rarest euarthropods of the extremely prolific Burgess Shale Lagerstätte. Even more problematic, the two species from the Wheeler strata of the House Range are represented by a single specimen each. Only the Tremadocian representative of the genus seems relatively common in some localities of the lower Fezouata Shale, possibly due to more suitable depositional (more proximal?) or environmental (shallower-water?) conditions.

Variability in the preservation of *Thelxiope* specimens may also be deceptive, as best exemplified by USNM114916 (Figs. 3B, 3C). This specimen of *T. palaeothalassia* is seemingly equipped with a single sagittal spine only, the hypertrophied pygidial one. We believe that this is due to the fact that the sagittal structures of the dorsal exoskeleton are covered by the surrounding sediment, the greater width (tr.) of the hypertrophied spine explaining why it is visible exsagittally. The way the fossils have been flattened also complicates morphological comparisons between different specimens and species. *Thelxiope* dorsal exoskeletons are always preserved flattened (mostly) laterally, which likely results from the presence of long sagittal protrusions on a subcylindrical body. Yet, the cephalic shields of most specimens show somewhat oblique orientations, as if they were slightly tilted ventrolaterally before flattening (Figs. 2, 3A and 6). The reason for that is unclear, but it might relate to the way the long cephalic appendages protruded from under the cephalic shield, as possibly exemplified by USNM424114 (Fig. 6). This varying degree of tilting of the cephalic shield impacts the accuracy of size comparisons between cephalic shield and pygidium of a given individual, and of cephalic shield shape comparisons between specimens, hence why we refrain from using such characters in the diagnoses.

The taxonomy of *Mollisonia* is similarly complicated by the scarcity of specimens and their inconsistent preservation. Although not as rare as *Thelxiope*, *Mollisonia* is known from only a handful of specimens in most deposits: six in the Kaili Formation (Zhang et al., 2002; Zhao et al., 2011), four in the Wheeler Formation in the House Range (Lerosey-Aubril et al., in press), and one each in the Dol-cyn-Afon Formation (Botting et al., 2015), the Spence Shale, and the Wheeler Formation in the Drum Mountains (Briggs et al., 2008). *Mollisonia* has as-yet been found by the dozens at two Burgess Shale localities only, the Walcott Quarry (Caron & Jackson, 2008) and Marble Canyon (Aria & Caron, 2019). The orientation of the specimens before flattening varies from perfectly lateral to dorsal (e.g., Zhang et al., 2002; Aria & Caron, 2019), probably because this orientation was not constrained by the presence of a row of prominent sagittal structures. Plastic deformation of the (partially decayed?) dorsal exoskeleton can also result in significant outline variations (Lerosey-Aubril et al., in press), and a lateral tilt of the cephalic region is seldom observed (e.g., Aria & Caron, 2019, Fig. 1E).

In summary, the complicated taxonomic history of *Mollisonia* and *Thelxiope* species is explained by a combination of scarce fossil material and variable preservation. Focusing on the characteristics of the sagittal spines may offer robustness to the taxonomic framework proposed herein for *Thelxiope*, at least until the discovery of more material reveals the discriminating potential of other morphological characteristics.

### The three-segmented pygidium of *Mollisonia* and *Thelxiope*

**Figure 9:**
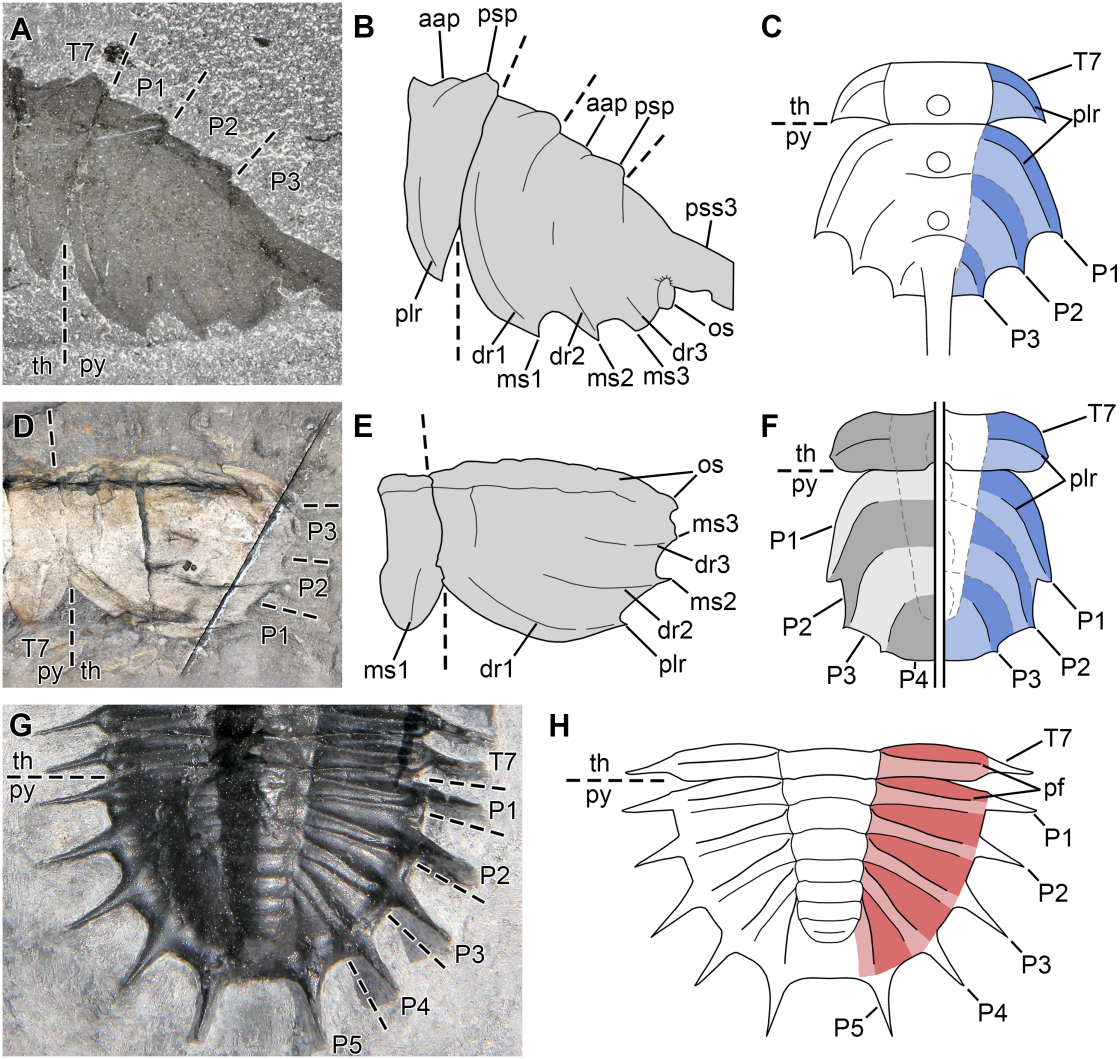
Comparison between the pygidial structures of Mollisoniidae and trilobites. (A–C) Pygidial structure in *Thelxiope*. (A, B) Picture and interpretative drawing, respectively, of specimen USNM144916. (C) Schematic interpretation of pygidial segmentation; the presence of dorsal ridges and marginal spines serially homologous to the tergopleural ridges and acute tips of thoracic tergopleurae indicates that the pygidium includes three trunk tergites. (D–F) Pygidial structure of *Mollisonia*. (D, E) Picture and interpretative drawing, respectively, of specimen USNM83951 (mirrored). (F) Pygidial segmentation following Aria & Caron’s (2019) interpretation (left) and the interpretation proposed herein (right). According to Aria & Caron (2019), the pygidium includes four tergites bounded by the (intersegmental) tergopleural ridges. In our opinion, the dorsal ridges and marginal spines are serially homologous to the tergopleural ridges and acute tips of thoracic tergopleurae and therefore intrasegmental structures; the fact that they are three pairs of each indicate a three-segmented pygidium. (G, H) Pygidial structure in the trilobite *Olenoides*. (G) Picture of specimen BPM1109 (Photo credit: Enrico Bonino). (H) Schematic interpretation of pygidial segmentation; similar (but not necessarily homologous) to the tergopleural ridges of Mollisoniidae, the pleural furrows of trilobites are intrasegmental structures, which separate each tergite pleura into anterior and posterior bands. They may be the only furrows visible on the pygidial pleural fields of some trilobites. Credit for all drawings: Rudy Lerosey-Aubril. Abbreviations: *aap*, anterior articulating part of axial ring; *dr*, dorsal ridge; *ms*, marginal spine; *os*, opposite side of pygidium; *P*, non-articulated pygidial tergite; *pf*, pleural furrow; *plr*, tergopleural ridge; *psp*, posterior swollen part of axial ring; *pss*, pygidial sagittal spine; *py*, pygidium; *T*, thoracic tergite; *th*, thorax.

*Simonetta (1964)* described the pygidium of *Thelxiope* as composed of three ‘fused’ tergites, each bearing a sagittal spine, plus a ‘telsonic’ spine. *T. holmani* sp. nov. demonstrates that only three spines project from the dorsal surface of the pygidium in *Thelxiope* (Fig. 4), amongst which the posteriormost one (‘telsonic’ spine of Simonetta, 1964) may be hypertrophied. These sagittal spines, like the marginal spines and the tergopleural ridges, evidence the three-segmented nature of this pygidium—this sclerite is made of three tergites, which are essentially larger, non-articulated versions of thoracic tergites. In *T. palaeothalassia* (Figs. 2 and 3), the structures of thoracic and pygidial tergites are strikingly similar: each comprises an axial region split into a flat anterior part and a raised posterior part bearing the sagittal spine, and tergopleural regions with ridges running towards acute distal tips/marginal ‘spines’ (Figs. 9A–9C). If the pygidium does include a telsonic (i.e., non-segmented) part, it is highly reduced and located posterior to the posteriormost sagittal spine, which is borne by the third pygidial segment. This observation is of phylogenetic significance, for it means that the third pygidial spine of *Thelxiope*—the hypertrophied one in *T. palaeothalassia*, *T. spinosa*, and *Thelxiope* sp. nov. A—is not comparable to the tailspine of *Habelia* (*contra* Simonetta & Delle Cave, 1975; Bousfield, 1995).

Close phylogenetic relationships between *Thelxiope* and *Habelia* were ruled out by *Aria & Caron (2017)*, who emphasized the significantly different trunk organization in the two taxa. The trunk of *Thelxiope* (and Mollisoniidae) comprises seven articulated tergites (thorax) and three non-articulated ones (pygidium), whereas that of *Habelia* is composed of 12 articulated tergites and a long tailspine (or ‘telson’; Aria & Caron, 2017). Spinosity patterns—a major reason for the original grouping of these two taxa within the family Habelliidae (Simonetta & Delle Cave, 1975)—also notably differ. In *Habelia*, the cephalic shield lacks a sagittal spine, the trunk bears paired dorsal spines that are only well-developed anteriorly, and the long posteriormost spine represents a distinct structure articulated with the rest of the body (the ‘telson’). By contrast, the spinosity pattern in *Thelxiope* consists in a single row of sagittal spines that extends from the posteriormost cephalic segment to the posteriormost pygidial one. Close affinities between these two genera are not supported either by the appendicular anatomy of *Mollisonia* (Aria & Caron, 2019), which shows a series of homonomous limbs (one pair per segment), all with strongly reduced (absent?) endopods, under the whole post-cephalic region. In *Habelia*, the anteriormost five trunk segments (‘thorax’) differs from the more posterior ones (‘post-thorax’) by the fact that they bear biramous appendages with particularly long endopods (Aria & Caron, 2017). In summary, the dorsal and ventral anatomy of *Habelia* documents a tagmosis irreconcilable with its assignment to Mollisoniidae. If *Habelia* and *Thelxiope* may both belong to the stem lineage of chelicerates, they remain distantly related taxa.

In essence, the pygidium of *Thelxiope* is simply a spinose version of the three-segmented pygidium of *Mollisonia* (Figs. 9D–9F). *Aria & Caron (2019)* described the pygidium of *Mollisonia* as resulting from the ‘fusion of the tergites of the four posteriormost segments’. Considering the putative incorporation of a thoracic tergite to the cephalic shield, they also argued for the ancestral presence of 12 segments in the trunk of *Mollisonia*, an organisation supposedly reflecting the ground pattern of Chelicerata (Aria & Caron, 2019; Legg, 2014; but see Dunlop & Lamsdell, 2017). We believe that Aria & Caron (2019) erroneously reconstructed the pygidial structure of *Mollisonia*, because of a misinterpretation of the tergopleural ridges as intersegmental, rather than intrasegmental structures. These authors regarded these ridges as marking the boundaries between pygidial segments (see their Fig. 2I and description in their supplementary information; left side of Fig. 9F), whereas our re-examination of *Thelxiope* demonstrates that they are instead serial homologues of the tergopleural ridges of the thoracic tergites, and therefore borne by the tergopleurae of the non-articulated pygidial tergites in the two sister-taxa (right side of Fig. 9F). Segment boundaries in Mollisoniidae may be expressed by faint furrows on the axial region (e.g., Zhang et al., 2002, fig. 1.1; Figs. 9A–9C), but not in the tergopleural regions; there, these segmental limits are suggested by the re-entrants of the posterolateral margins only. An interesting parallel can be drawn with the pleural furrows of trilobite pygidia (Figs. 9G, 9H), serial homologues of the thoracic pleural furrows that are typically more deeply incised than the interpleural furrows (the latter being the serial homologues of the thoracic pleural articulations) (Whittington et al., 1997, p. 62, 63). As in *Thelxiope*, it is unclear whether the posteromedian part of the pygidial tergopleural field (i.e., the post-axial field) in *Mollisonia* includes a telsonic part; this area might just as well represent the fused tergopleural regions of the third pygidial tergite (Figs. 9C and right side of  9F). Lastly, Aria & Caron’s (2019) description of four (rather than three) pairs of pygidial appendages is to us not convincingly supported by the sole illustrated specimen (a specimen of *M. symmetrica*; their extended data Fig. 4), and was likely influenced by the misinterpretation of the pygidial segmentation. Three pairs of pygidial appendages appear more likely to us considering the three-segmented nature of the pygidium, as well as the observation of only three nerve ganglia in the pygidial region of a *Mollisonia* specimen (work in progress).

## Conclusions

*Thelxiope* primarily differs from other mollisoniids by the presence of well-developed sagittal spines all along the dorsal exoskeleton. The characteristics of these spines allow the recognition of four distinct species. Three of them represent particularly rare components of Miaolingian (Wuliuan–Drumian) exceptionally-preserved faunas from Laurentia, whereas the fourth appears to be relatively common in the Tremadocian lower Fezouata Shale of Morocco (West Gondwana). The scarcity and inconsistent preservation of most *Thelxiope* materials to date prevent the use of other morphological features for the systematics of this taxon. The pygidium in both *Thelxiope* and *Mollisonia* consists of three non-articulated tergites comparable in structure to thoracic tergites. This suggests that this caudal shield does not represent a distinct tagma, and may be better regarded as a ‘frozen growth zone’ like the pygidia of most trilobites (Minelli, Fusco & Hughes, 2003; Hughes, 2003). Our current understanding of the appendicular anatomy of *Mollisonia* supports the view of a trunk forming a single tagma, since it seems associated with a series of homonomous limbs all along (Aria & Caron, 2019). Investigating the ontogenies of representatives known from abundant materials (e.g., *Mollisonia* from Marble Canyon, *Thelxiope* from the Fezouata) could help determine whether the generation of trunk segments and their allocation into two trunk pseudotagmata (*sensu* Lamsdell, 2013) in mollisoniids follow modalities comparable to those described in trilobites: (1) generation from a posteriorly located growth zone; (2) migration anteriorly as new segments are added posteriorly; (3) and ultimately, release in the thorax anteriorly via the formation of an articulation (Hughes, 2003). Such comparisons could provide critical new arguments as to whether mollisoniids belong to Artiopoda (Legg, Sutton & Edgecombe, 2013), in which the presence of a pygidium is a plesiomorphic character, or represent atypical chelicerates that independently evolved a pygidium (Aria & Caron, 2019).

## Additional Information and Declarations

### Data Availability

The following information was supplied regarding data availability: The fossils studied, illustrated and/or discussed in this article are available in the collections of: the Back to the Past Museum, Cancun, Mexico (BPM1019); the Geological Survey of Canada, Ottawa, Canada (GSC74990; GSC78462); the Museum of Comparative Zoology at Harvard University, Cambridge, USA (MCZ197957); the Smithsonian National Museum of Natural History, Washington, USA (USNM83951, USNM144914, USNM144915, USNM144916, USNM424114); and the Yale University Peabody Museum, New Haven, USA (YPM-IP226544).

### New Species Registration

The following information was supplied regarding the registration of a newly described species: Publication LSID: urn:lsid:zoobank.org:pub:304BC80B-F898-4842-ACBC-BE3F692CDAE4. Thelxiope holmani: urn:lsid:zoobank.org:act:097471F6-DB92-4356-BF02-C50EFA363808
